# Self-assembly Synthesis of Molecularly Imprinted Polymers for the Ultrasensitive Electrochemical Determination of Testosterone

**DOI:** 10.3390/bios10030016

**Published:** 2020-02-27

**Authors:** Kai-Hsi Liu, Danny O’Hare, James L. Thomas, Han-Zhang Guo, Chien-Hsin Yang, Mei-Hwa Lee

**Affiliations:** 1 Department of Internal Medicine, Division of Cardiology, Zuoying Branch of Kaohsiung Armed Forces General Hospital, Kaohsiung 813, Taiwan; liukaihsi@gmail.com; 2 Department of Chemical and Materials Engineering, National University of Kaohsiung, Kaohsiung 81148, Taiwan; pzps0964431@gmail.com; 3 Department of Bioengineering, Imperial College, London SW7 2BY, UK; d.ohare@imperial.ac.uk; 4 Department of Physics and Astronomy, University of New Mexico, Albuquerque, NM 87131, USA; jthomas@unm.edu; 5 Department of Materials Science and Engineering, I-Shou University, Kaohsiung 84001, Taiwan

**Keywords:** testosterone, molecular imprinting, electronically conductive polymer, electrochemical sensing, urine

## Abstract

Molecularly imprinted polymers (MIPs) can often bind target molecules with high selectivity and specificity. When used as MIPs, conductive polymers may have unique binding capabilities; they often contain aromatic rings and functional groups, which can undergo π-π and hydrogen bonding interactions with similarly structured target (or template) molecules. In this work, an electrochemical method was used to optimize the synthetic self-assembly of poly(aniline-*co*-metanilic acid) and testosterone, forming testosterone-imprinted electronically conductive polymers (TIECPs) on sensing electrodes. The linear sensing range for testosterone was from 0.1 to 100 pg/mL, and the limit of detection was as low as ~pM. Random urine samples were collected and diluted 1000-fold to measure testosterone concentration using the above TIECP sensors; results were compared with a commercial ARCHITECT ci 8200 system. The testosterone concentrations in the tested samples were in the range of 0.33 ± 0.09 to 9.13 ± 1.33 ng/mL. The mean accuracy of the TIECP-coated sensors was 90.3 ± 7.0%.

## 1. Introduction

For men beyond the age of 30, testosterone levels gradually decline with increasing age [1]. Some possible causes of low testosterone levels are testicular injury or infection [2], dysfunctional hormone excretion, medication, inflammation and chronic illness (such as chronic kidney failure [3], dysthymic disorder [4], alcoholism, hepatic cirrhosis [5] or obesity [6]). A homecare system, monitoring testosterone concentration, may offer important diagnostic benefits.

Molecularly imprinted polymers (MIPs) for measuring the concentration of hormones and metabolites have been produced in the last decade and used for *optical sensing* by surface plasmon resonance (SPR) to detect progesterone [7], cholesterol [7] and testosterone [7,8,9,10]. More traditional laboratory-based methods have also been used, such as: gas chromatography (GC) for measurement of anabolic steroids [11] and extraction steroids [12]; liquid chromatography (LC) for measurement of epitestosterone [13], testosterone [13,14,15], or other steroids [16]; capillary electrophoresis (EC) linked with mass spectrometry (MS) for testosterone [17], epitestosterone [17], urinary steroid hormones [18] and estrogenic endocrine disruptors [19]; diode-array detection (DAD) of steroids [20], progesterone [21] and testosterone [21] in human urine [11,17,20,21,22,23] or in goat milk [24]. The functional and crosslinking monomers that have been used in molecular imprinting include acrylamide [16], methacrylic acid (MAA) [7,8,9,10,11,13,15,17,20,21,24,25,26,27,28], trifluoromethacrylic acid (TFMAA) [13], 2-hydroxyethyl methacrylate (HEMA) [9,26], ethylvinylbenzene (EVB) [7], 2-vinylpyridine (2VP) [12], 4-vinylpyridine (4VP) [12,20,26], dopamine (DA) [14]; divinylbenzene (DVB) [7,13], ethylene glycol dimethacrylate (EGDMA) [8,9,10,11,16,17,20,21,22,23,24,26,27,28], trimethylolpropanetrimethacrylate (TRIM) [11,20,21], pentaerythritol triacrylate (PETRA) [22] and 5α-androstane-3α, 17β-dimethacryloxy ester (AnDMA) [13]. Synthetic functional monomers such as (1) 1-(4-vinylphenyl)-3-(3,5-bis(trifluromethyl)phenyl)urea (FM1) and 1-benzyl-3-vinyl- 2,3-dihydro-1H-imidazolium bromide (FM2) have been used to isolate testosterone glucuronide [22] and (2) the bifunctional cross-linker N,O-bismethacryloylethanolamine (NOBE) have been synthesized to form patterned MIP structures to detect testosterone in buffer, urine and saliva using electrochemical impedance spectroscopy (EIS) [29]. A visible light-activated photo-iniferter agent, 4-cyano-4-[(dodecylsulfanyl- thiocarbonyl) sulfanyl]pentanoic acid (CDTPA), was employed for chain extension with poly(ethylene glycol methacrylate phosphate) brushes by reversible addition–fragmentation chain transfer (RAFT) polymerization [30]. In addition, the imprinting of other steroid hormones (e.g., 17β-estradiol) has been used for increasing the retention of testosterone in solid phase extraction [18]. A recent comparison of methods for testosterone determination reported limits of detection (LODs) in the range of 0.08–20.0 ng/mL [14]. The majority of techniques employed either SPR or chromatographic techniques; electrochemical methods, despite their advantages of low cost and flexibility, have not been reported for testosterone.

The use of conducting polymers in sensor-related technology [31] and electrochemically prepared MIPs have each been reviewed [32,33]. Polyaniline (PANI) derivatives can be electrochemically polymerized [34,35], chemically polymerized [36], and even simultaneously self-assembled/polymerized [37,38] in aqueous solutions. PANI derivatives have attracted substantial scientific interest in recent decades owing to their favorable combination of characteristics, including: a more diverse structure and better thermal and radiation stability than polypyrrole; lower cost than polythiophene; ease of synthesis; and moderately high conductivity. They have, therefore, been used in a wide range of applications [39], such as micro-electronics, corrosion protection, battery electrodes, and sensors [40].

In this work, an electrochemical method was employed to optimize the synthetic self-assembly of poly(aniline-*co*-metanilic acid) and template molecules by coating on the sensing electrodes, to form testosterone-imprinted polymers (TIECPs). Generally, the polymerization of polyaniline needs to be carried out in an acidic environment. In this environment, it is often necessary to add an extra inorganic/organic acid as a dopant which provides hydrogen ions to dope the amine groups on polyaniline. The resulting doped polyaniline has moderately high conductivity, an important attribute for electrochemical sensing applications. In this study, aminobenezenesulfonic acid (metanilic acid) has a dual role as a reactive monomer (aminobenzene) and as a dopant (sulfonic acid group); metanilic acid was copolymerized with aniline monomer to obtain self-doped polyaniline films, eliminating the need for an extraneous acid. The TIECPs were characterized by their imprinting effectiveness (α), which is the ratio of current densities generated in the sensing of template molecules by imprinted and non-imprinted polymer-coated electrodes. The surface morphologies and electronic spectra of the TIECPs during self-assembly were obtained using a scanning electron microscope (SEM). Finally, random urine samples were collected, and their testosterone concentrations were measured using TIECP sensors. The experimental results were compared with results from a commercial ARCHITECT ci 8200 system to confirm the reliability.

## 2. Materials and Methods

### 2.1. Reagents

Aniline (ANI, Merck, Darmstadt, Germany) was distilled under reduced pressure, and metanilic acid or *m*-aminobenzenesulfonic acid (MSAN, Acros Organics, Geel, Belgium) was purified by recrystallization twice from deionized (DI) water. Testosterone (≥98.0%), progesterone (≥99.0%), urea (minimum ≥98.0%), creatinine (minimum ≥99.0%), and ethanol were purchased from Sigma-Aldrich Co. (St. Louis, MO, USA). 17β-Estradiol (≥98.0%) was from Alfa-Aesar (Ward Hill, MA, USA). Ammonium peroxydisulfate (APS) used as the initiator was from Wako (Osaka, Japan). The ITO-coated glass substrates (~10 Ω·cm^−2^) were from Merck. Deionized water (18.2 MΩ), produced by a PURELAB Ultra (ELGA, High Wycombe, UK), was used in the preparation of buffers and for rinse solutions. All chemicals were used as received unless otherwise mentioned.

### 2.2. Synthesis and Characterization of Testosterone-imprinted Electronically Conductive Polymer (TIECP) films

The synthetic procedure has been successfully used in our previous studies. The surface of an ITO electrode was sequentially cleaned by using isopropanol, acetone, and distilled water before polymerization [41,42]. Electrically conductive polymer films were assembled (polymerized) on ITO glass (Merck, 1 × 1 cm^2^). Aniline (ANI) and *m*-aminobenzenesulfonic acid (MSAN) in mole ratios from 20–80% were dissolved in DI water, keeping the total amino group concentration at 57 mM. To make imprinted films, testosterone, employed as the template and target molecule in this study, was included at concentrations up to 100 μg/mL. Initiator/oxidant (APS, 0.5% (w/w)) was then added to the ANI/MSAN mixture, and polymerization proceeded by immersion of the ITO electrode in the monomer mixture at 25 °C. The APS acts as an oxidant resulting in copolymerization to form electron-conducting poly(aniline-*co*-metanilic acid) from the aqueous mixture of ANI and MSAN [38]. Finally, ethanol solution (5% v/v) was employed for the removal of target molecules.

### 2.3. Electrochemical Characterization of TIECP-coated Electrodes

The TIECP-coated electrode, counter electrode (Pt wire), and Ag/AgCl (Matsusada Precision, Japan) reference electrode were placed in a mixture solution including 20 μL sample (e.g., testosterone, urea, creatinine, 17β-estradiol and progesterone) and 20 mL 125 mM KCl, 5mM K_4_Fe(CN)_6_ and 5 mM K_3_Fe(CN)_6_ solution; the cyclic voltammetry of the electrochemical reactions was performed using a potentiostat (608-1A, CH Instruments, Inc., Austin, TX). The film thickness of the electroactive film can be directly quantitatively measured from the current density of the redox couple using the calibration curve between the current density and film thickness [41,42]. The potential was scanned from −0.3 V to 0.8 V at a san rate of 0.1 V/s [43,44] and the effects of target and interferent molecules (e.g., urea, creatinine, 17β-estradiol and progesterone) on the peak currents for the ferri-/ferrocyanide system were also recorded. TIECP films were freeze-dried before examination by a scanning electron microscope (Hitachi S4800, Hitachi High-Technologies Co., Tokyo, Japan), and atomic force microscopy (Solver P47H-PRO, NT-MDT Moscow, Russia) and a golden silicon cantilever (NSG01, NT-MDT).

### 2.4. The Determination of Testosterone in Human Urine Samples

Urine samples were collected from colleagues of the authors 4 h before the test, and diluted 1000-fold with 125 mM KCl, 5mM K_4_Fe(CN)_6_ and 5 mM K_3_Fe(CN)_6_ solution. The urine sample (1 mL) was stored in an Eppendorf microcentrifuge tube at 4 ^o^C and analyzed for testosterone with the ARCHITECT *ci* 8200 system (Abbott Laboratories, Abbott Park, Illinois, USA.). The Abbott Architect ci8200 analyzer was specifically designed to provide clinical chemistry and immunoassay testing, which combines immunoassay and clinical chemistry on one integrated platform and runs up to 200 immunoassay tests and up to 1200 clinical chemistry tests an hour. Please refer to the following website for further information: https://www.corelaboratory.abbott/int/en/offerings/brands/architect/architect-ci8200.

## 3. Results and Discussion

**Figure 1:**
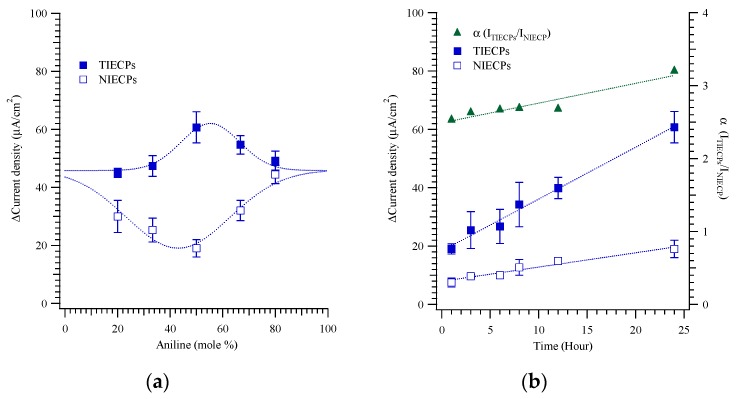
(**a**) Aniline concentration and changes in current density from cyclic voltammograms (CVs) in a solution of 20 mM potassium ferricyanide (K_3_[Fe(CN)_6_]), 20 mM potassium ferrocyanide (K_4_[Fe(CN)_6_]), and 0.5 M KCl with/without 10 pg mL^−1^ of testosterone on testosterone- and non-imprinted poly(aniline-*co*-metanilic acid)-coated electrodes, (**b**) imprinting effectiveness and current density versus polymerization duration of testosterone-imprinted and non-imprinted poly(aniline-*co*-metanilic acid).

To assess the performance of testosterone-imprinted electrically conductive polymers (TIECPs) and non-imprinted electrically conductive polymers (NIECPs), current density for the ferri-ferrocyanide redox couple was measured in the presence and absence of 10 pg/mL testosterone. The current density difference (with and without testosterone) is plotted in Figure 1, for both imprinted TIECPs and NIECPs, as a function of composition. As the mole ratio of ANI to MSAN was varied, the largest difference between the testosterone responses for TIECPs and for NIECPs was obtained at 50 mole % aniline (a 1:1 composition). Interestingly, not only was the electrochemical response of imprinted electrodes maximized at this composition, but the response of non-imprinted electrodes was also minimized, as discussed below. For this composition, the current density differences were 60.70 ± 5.37 and 19.00 ± 3.00 μA/cm^2^ for TIECPs and NIECPs, respectively. This corresponds to an imprinting effectiveness of slightly over 3.

In the ANI/MSAN copolymers, the sulfonic group is likely to bind to the secondary amine of aniline, and, thus, to be unavailable to bind testosterone. The effect should be most pronounced at equimolar ratios, and, in agreement with this expectation, the response of non-imprinted polymers to testosterone is minimized at equimolar composition. Interestingly, the response of the imprinted polymers increases at equimolar composition, in spite of the potential for reduced hydrogen bonding to the target. This may be caused by increased stiffness of the matrix, improving the binding site shape recognition, and the electrochemical reaction may occur only on the electrode surface. (In addition, of course, hydrogen bonding in the binding sites may still occur, as the hydrogen-bonded template will effectively sequester the sulfonic acid and prevent it from binding to ANI). Figure 1b shows that current density differences grow with increased polymerization time. Nonetheless, imprinting effectiveness varies only weakly with polymerization time.

**Figure 2:**
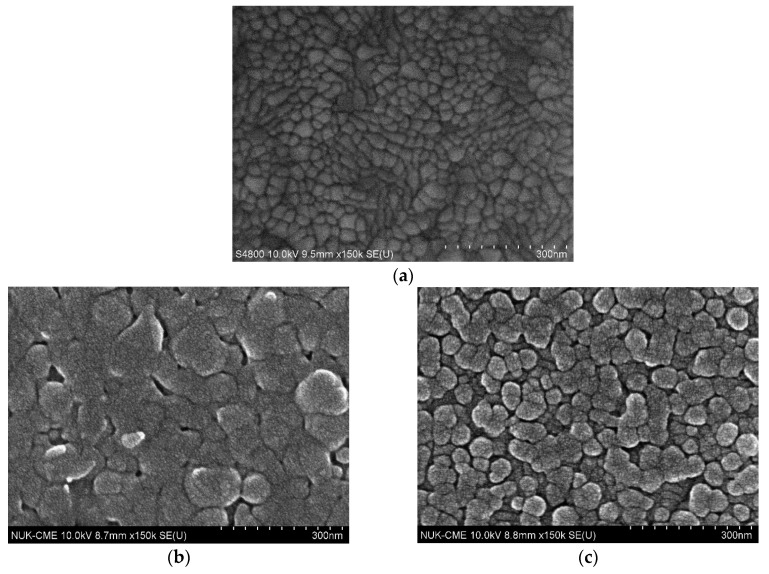

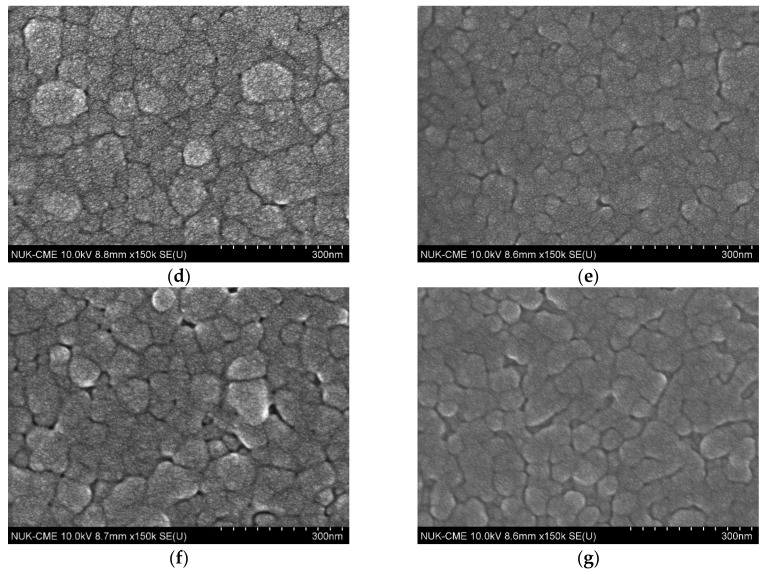
Scanning electronic microscopy images. (**a**) bare ITO glass; (**b**–**g**) testosterone-imprinted (**left**) and non-imprinted (**right**) poly(aniline-co-metanilic acid) containing 50 mole % of aniline: (**b**,**c**) before washing; (**d**,**e**) after washing; (**f**,**g**) after washing (template removed). The washed, imprinted films show considerable surface roughness, presumably owing to the presence of numerous binding sites or cavities.

**Figure 3:**
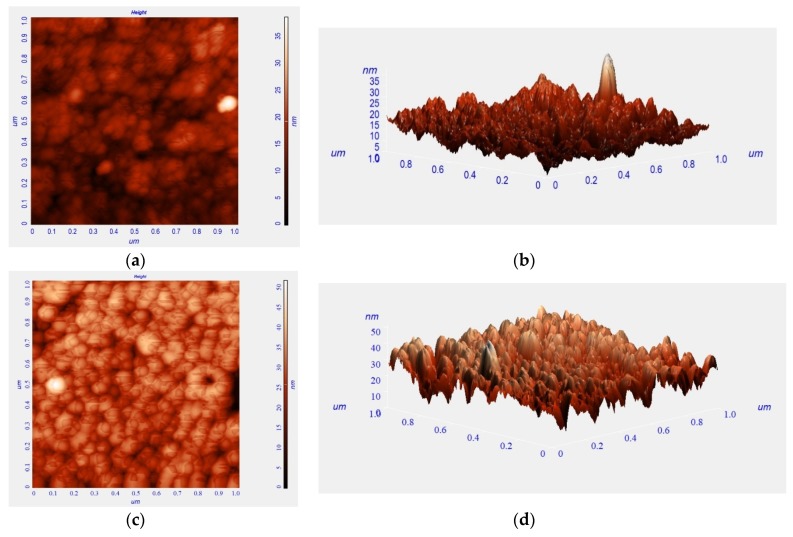

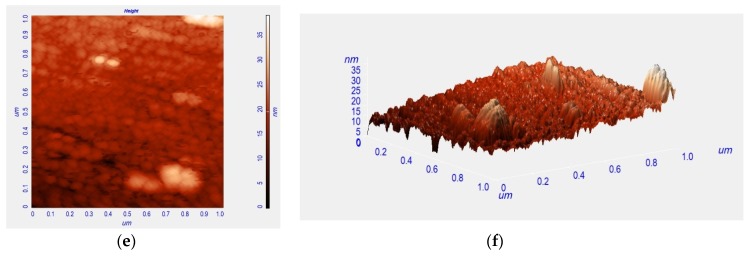
AFM images of a TIECP-coated electrode (**a**,**b**) before, (**c**,**d**) after template removal and (**e**,**f**) rebinding of the target molecules. (**left**) Height shown as intensity; (**right**) 3D representation (height exaggerated relative to horizontal scale.).

Figure 2 displays scanning electron microscope images including bare ITO glass, TIECPs and NIECPs before and after template removal, as well as after binding with target molecules. In Figure 2a, the grain size of bare ITO glass was from 15 nm to 80 nm; the morphologies and grain sizes of ITO were different from TIECPs and NIECPs thin films. Many poly(ANI-*co*-MSAN) particles and aggregates were observed on the surface of the sensing electrodes. The sizes of the aggregates were about 60–150 and 30–60 nm on the surface of TIECPs and NIECPs, respectively. SEM images of TIECP and NIECP electrodes rinsed in 5% ethanol, shown in Figure 2d,e, reveal that the unbound poly(ANI-*co*-MSAN) particles on the surfaces had been cleared out. Based on the SEM images, the surface morphologies of the TIECP and NIECP thin films following readsorption of 10 pg/mL of testosterone are less rough after readsorption (compare Figure 2f,g to Figure 2d,e respectively), when the cavities on the poly(ANI-*co*-MSAN) particle surface were refilled with the target molecules. The washed, imprinted films show considerable surface roughness, presumably owing to the presence of numerous binding sites or cavities. Further details of the surface morphology of TIECPs, determined by AFM, are shown in Figure 3; the size of polymer particles agreed with SEM images. The average surface roughness increased from 3.3 nm to 4.8 nm when template was removed, and then decreased to 3.4 nm upon rebinding of the target molecules.

**Figure 4:**
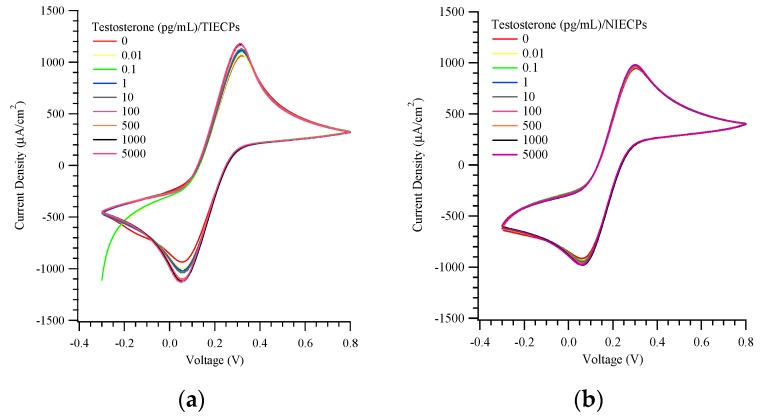
Cyclic voltammograms of various target concentrations on (**a**) testosterone- (TIECPs) and (**b**) non-imprinted poly(aniline-co-metanilic acid) (NIECPs) coated electrodes.

Cyclic voltammetric sensing of testosterone at concentrations of 0.01 to 5000 pg/mL was conducted using the TIECP and NIECP-coated electrodes, as presented in Figure 4a,b. The pH value was nearly constant at the different concentrations of testosterone. The maximum current densities of MIPs and NIECPs electrodes were ca. 1200 and 1000 μA/cm^2^, respectively. The electrochemical current densities were approximately 30-fold higher than our earlier work on MIPs using imprinted poly(ethylene-*co*-vinyl alcohol) (EVAL). (EVAL has been successfully used to measure small molecules, such as creatinine and urea [43]).

**Figure 5:**
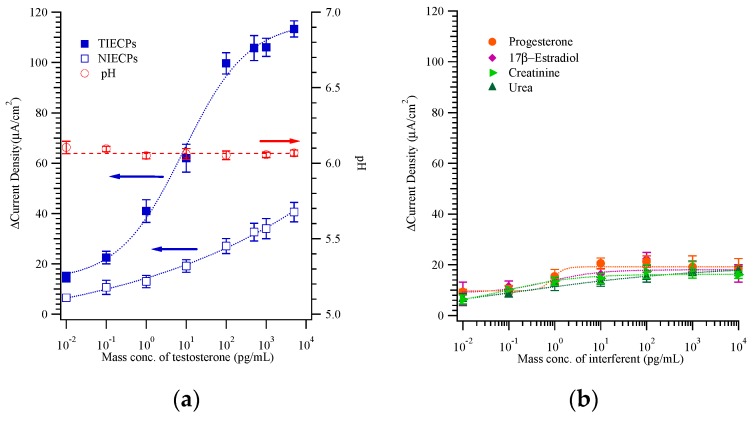
(**a**) The calibration curve of oxidation-peak current density and pH value from CVs against mass concentrations of testosterone on molecularly and non-imprinted poly(aniline-*co*-metanilic acid) based sensors. (**b**) The effect of the interferents (e.g., 17β-estradiol (diamonds), progesterone (circles), urea (triangles), and creatinine (right triangles)) on peak current response was tested and is shown (n > 3).

Figure 5a shows the changes in oxidation-peak current density for a range of testosterone (mass) concentrations, for both imprinted and non-imprinted polymeric sensors; also shown is the pH of the samples, which all fell between 6.12 ± 0.05 to 6.07 ± 0.02 for testosterone mass concentrations from 0 to 500 pg/mL. Thus, the mass concentration of testosterone had essentially no effect on the pH value of these buffered solutions.

Figure 5a also shows that the useful linear dynamic range is 0.1 to 100 pg/mL for testosterone, with the signal beginning to saturate at the higher concentration. We compare these results to other testosterone detection methods: micro-patterned MIPs on functionalized diamond-coated substrates were reported to show linearity from 0.5 to 20 nM testosterone [29]; reflectance spectra of TIECPs films showed a shift of the Bragg diffraction peak that correlated with testosterone concentration in the range 5–100 ng/mL [15]. Thus, the electrochemical approach presented here is highly sensitive.

Figure 5b shows that the current densities caused by potential interferents found in real urine samples (including 17β-estradiol, progesterone, urea, and creatinine) were less than 20 μA/cm^2^, quite similar to the current density caused by testosterone on non-imprinted sensors. Thus, the testosterone-imprinted poly(ANI-*co*-MSAN) film is effectively *non-imprinted* for these potential interferents. The selectivity of MIPs that were prepared by polymerization in this study exceeded that achieved in our previous work based on phase inversion [32]; for example, the 5-fold preference for testosterone over other interferents (in this work), vs. about a 2-fold preference for urea over creatinine in [32].

Finally, Table 1 summarizes analyses of random urine samples performed by using the ARCHITECT *ci* 8200 system. The concentrations of testosterone in the samples fell in the range of 0.33 ± 0.09 to 9.13 ± 1.33 ng/mL. The current deviations measured by the TIECP sensor in at least three urine samples ranged from 27.35 ± 1.15 to 65.15 ± 2.95 μA/cm^2^ corresponding to concentrations of 0.28 ± 0.07 to 8.99 ± 2.68 ng/mL (standard deviations of at least three individual measurements). The mean accuracy of TIECPs-coated sensors was 90.3 ± 7.0%. Note that the accuracy was slightly lower, approximately 80–85%, when the testosterone concentration in urine was less than about 2.0 ng/mL.

## 4. Conclusions

Monomers bearing an aromatic ring may have a greater tendency than other aliphatic structures to exhibit hydrogen bonding interactions with target (or template) molecules [32], making them attractive for use in molecularly imprinted polymer applications. Our experiments showed these materials are especially suitable for the preparation of molecularly imprinted polymers for steroid hormones, by demonstrating the specific recognition of testosterone by imprinted ANI/MSAN copolymers. This work also demonstrated the importance of monomer ratio in creating films with specific and selective recognition, and detailed the distinctive surface morphologies of both imprinted and non-imprinted films for effective and accurate electrochemical detection of testosterone in urine.

## Figures and Tables

**Table 1:** Comparison of real sample measurement by ARCHITECT ci 8200 system and the TIECP sensors.

Sample No.	ARCHITECT *ci* 8200 System Testosterone (ng/mL)	TIECP Sensors		Accuracy (%)
		ΔCurrent (μA/cm^2^)	Avg. conc. (ng/mL)	
1	0.79 ± 0.02	33.85 ± 0.25	0.64 ± 0.03	81.0
2	1.51 ± 0.08	40.65 ± 0.75	1.28 ± 0.13	84.8
3	2.32 ± 0.01	47.10 ± 1.90	2.27 ± 0.50	97.8
4	0.33 ± 0.09	27.35 ± 1.15	0.28 ± 0.07	84.8
5	3.04 ± 0.18	50.05 ± 1.95	2.88 ± 0.62	94.7
6	9.13 ± 1.33	65.15 ± 2.95	8.99 ± 2.68	98.5
